# Exploring the Pivotal Immunomodulatory and Anti-Inflammatory Potentials of Glycyrrhizic and Glycyrrhetinic Acids

**DOI:** 10.1155/2021/6699560

**Published:** 2021-01-07

**Authors:** Seidu A. Richard

**Affiliations:** Department of Medicine, Princefield University, P. O. Box MA 128, Ho, Ghana

## Abstract

Licorice extract is a Chinese herbal medication most often used as a demulcent or elixir. The extract usually consists of many components but the key ingredients are glycyrrhizic (GL) and glycyrrhetinic acid (GA). GL and GA function as potent antioxidants, anti-inflammatory, antiviral, antitumor agents, and immuneregulators. GL and GA have potent activities against hepatitis A, B, and C viruses, human immunodeficiency virus type 1, vesicular stomatitis virus, herpes simplex virus, influenza A, severe acute respiratory syndrome-related coronavirus, respiratory syncytial virus, vaccinia virus, and arboviruses. Also, GA was observed to be of therapeutic valve in human enterovirus 71, which was recognized as the utmost regular virus responsible for hand, foot, and mouth disease. The anti-inflammatory mechanism of GL and GA is realized via cytokines like interferon-*γ*, tumor necrotizing factor-*α*, interleukin- (IL-) 1*β*, IL-4, IL-5, IL-6, IL-8, IL-10, IL-12, and IL-17. They also modulate anti-inflammatory mechanisms like intercellular cell adhesion molecule 1 and P-selectin, enzymes like inducible nitric oxide synthase (iNOS), and transcription factors such as nuclear factor-kappa B, signal transducer and activator of transcription- (STAT-) 3, and STAT-6. Furthermore, DCs treated with GL were capable of influencing T-cell differentiation toward Th1 subset. Moreover, GA is capable of blocking prostaglandin-E2 synthesis via blockade of cyclooxygenase- (COX-) 2 resulting in concurrent augmentation nitric oxide production through the enhancement of iNOS2 mRNA secretion in Leishmania-infected macrophages. GA is capable of inhibiting toll-like receptors as well as high-mobility group box 1.

## 1. Introduction

Licorice extract is a Chinese herbal medication most often used as a demulcent or elixir [1]. Glycyrrhizin (GL) is one of the principally effective and efficient ingredients of licorice extract [1–3]. GL is a triterpene saponin which has aglycone component known as glycyrrhetinic acid (GA) [1]. GA is a pentacyclic triterpenoid of oleanene type with a hydroxyl group at C-3, a carboxyl moiety at C-30 as well as a ketone functional group at C-11 [2]. GL and GA have been demonstrated to possess antioxidant properties as well as robust anti-inflammatory, antiviral, antitumor, and immuneregulatory properties [4–6]. GL was capable of triggering the blockade of receptor-mediated endocytosis resulting in the inhibition of viral infiltration into the cells [5, 7].

GL triggers biological activities at the cellular level via novel gbPs, which are responsible for anti-inflammatory and antiviral effects [5, 8]. GL was capable of triggering the production of interferons (IFNs), accelerated the activities of natural killer (NK) cells as well as regulated the growth response of lymphocytes via the acceleration of interleukins- (IL-) 2 production [1, 8, 9]. Furthermore, GL has the ability to modulate the immune response at the initial stage of the disease process via the dendritic cells (DCs) [10]. GA inhibited anti-FAS antibody-triggered mouse liver injury but did not facilitate the upregulation of tumor necrotizing factor-*α* (TNF-*α*) messenger RNA (mRNA) secretion in the liver [11].

This review explores the fundamental immune and inflammatory players regulated by GL and GA. The “boolean logic” was utilized to search for the article on the subject matter. Most of the articles were indexed in PubMed with strict inclusion criteria being *in vitro* and *in vivo* up or downregulation of these immune and inflammatory biomarkers in diverse disease conditions. Inflammation, DCs, cyclooxygenase, and prostaglandins, cytokines like ILs, IFNs, TNF-*α*, nuclear factor-*κ*B (NF-*κ*B), mitogen-activated protein kinase (MAPK), Toll-like receptors (TLRs), high-mobility group box 1 (HMGB1), and chemokines like CCL11 as known as eotaxin 1 as well as enzymes like nitric oxide were explored.

## 2. Uses

Glycyrrhizin (GL) obtained from the dried roots of the licorice shrub is very sweet tasting and has been utilized as flavors in diverse food products and treatment of diseases for over 4000 years [12]. Currently, GL is used to flavor consumable products like chocolate, chewing gum, some alcoholic beverages, and cigarettes [12, 13]. Carbenoxolone (GC), the derivative of glycyrrhetinic acid (3*β*-11-oxoolean-12-en-30-oic acid 3-hemisuccinate), was used to treat peptic ulcer disease, allergic diseases, tumors or cancers, divers' viral diseases, and premenstrual syndromes [4–6]. They possess anti-inflammatory, antioxidant, antihyperglycemic, antilipidemic, and hepatoprotective properties [4–6]. Their key therapeutic usage of GC is for the treatment of viral diseases [14]. Chronic hepatitis C is the current target for use of GC in modern medicine [12, 15].

Several *in vivo* and *in vitro* studies showed that GL and GA have potent activities against hepatitis A, B, and C viruses, human immunodeficiency virus (HIV) type 1, vesicular stomatitis virus, herpes simplex virus, influenza A, severe acute respiratory syndrome- (SARS-) related coronavirus, respiratory syncytial virus, vaccinia virus, and arboviruses [2, 7, 16–20]. Also, GA was observed to be of therapeutic valve in human enterovirus 71, which was recognized as the utmost regular virus responsible for the hand, foot, and mouth diseases [2]. GL and GA demonstrated to have antibacterial actions against gram-positive bacteria like Bacillus subtilis and Staphylococcus aureus as well as gram-negative bacteria like Escherichia coli and Pseudomonas aeruginosa [2, 21, 22]. Furthermore, GA was capable of blocking the survival of methicillin-resistant S. aureus via the attenuation of its virulence gene expression [2, 23]. Also, GA has demonstrated to have antiparasitic potentials and its efficacy as an anti-malarial as well as antileishmanial has been elaborated in experimental studies [2, 24, 25].

## 3. Pharmacokinetics

GA is rapidly absorbed after oral administration, and its kinetics exhibited a biphasic association with a distribution phase preceded by a slower elimination phase [12, 26]. The medication is usually in a capsule form containing 500 mg of pure GA per capsule [26]. It was established that neither absorption nor elimination of GA was dose-dependent [26]. Several studies detected GA in both rats as well as human plasma [12]. On the other hand, GL is metabolized presystemically via commercial bacteria into GA and totally absorbed into the blood stream after oral intake [12, 27].

Studies have shown that the hydrolysis of GL to GA was carried out by bacteria strains like Eubacterium sp. (strain GHL), Ruminococcus sp. (PO1–3), and Clostridium innocuum (ES2406). These commercial bacteria were isolated from human feces and demonstrated enough hydrolyzing activity for GL [12, 28, 29]. The bacteria strains capable of hydrolyzing GL into GA possess a specific *β*-glucuronidase, because common *β*-glucuronidases like Escherichia coli were unable to hydrolyze GL [12, 28]. After hydrolysis of GL into GA, intestinal bacteria convert GA partially into 3-*α*-18*β*-GA, through a metabolic intermediary 3-oxo-18*β*-GA [12, 27].

Also, the plasma clearance of GL after an intravenous bolus dose to rats exhibited a biphasic pattern, in which the distribution phase was preceded by a slower elimination phase [30, 31]. However, realistic plasma levels of GA were observed to be approximately 100 mg/ml after intravenous administration [30, 31]. Also, the distribution of GA to the body tissues was negligible because tissue-to-blood partition coefficients were observed to <1 for all body tissues of rats [12, 32]. Interestingly, the uptake of GL into rat hepatocytes was competitively blocked by GA (46). This means that the plasma to liver transport of GA is facilitated by the same uptake carrier [31].

Studies have demonstrated that habitual usage of GA in consumable products may lead to adverse effects [12, 33]. It was established that capacity-regulated activities facilitate the metabolism, sinusoidal, and canalicular transport of GL [12]. Furthermore, GL was hydrolyzed by glucuronidases into 18-*β*-GA monoglucuronide in lysozymes of both rodents and humans [12]. This process may ultimately lead to edema, hypertension, and symptoms associated with electrolyte imbalances [12, 34].

## 4. Inflammation

**Figure 1:**
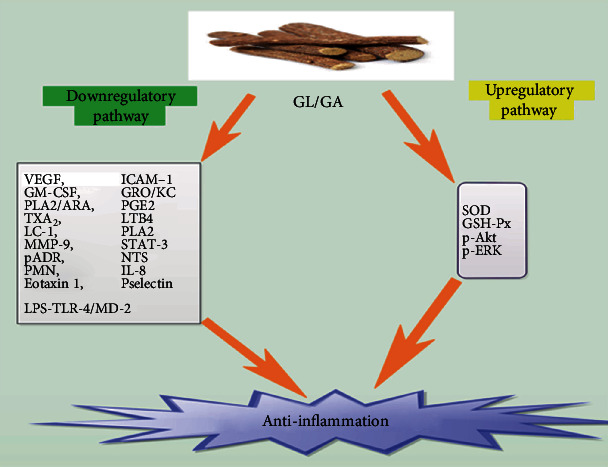
Shows a comprehensive down and upregulatory pathways via which GL and GA elicits anti-inflammation.

The fundamental processes involved in the eradication of threats posed to the host to organisms like bacterial and viral infections are the triggering of an acute inflammatory response [35]. Studies have shown that GL was capable of binding directly to lipoxygenase resulting in the generation of inflammatory mediators [36–38]. Also, GL selectively blocked the triggering of phosphorylation of these inflammatory mediators, which are mainly enzymes [36–38]. Specifically, GL as well as its derivatives was capable of blocking the generation of inflammatory chemokines like IL-8 and eotaxin 1, which are both powerful chemo-attractants to leukocytes during inflammation (Figure 1) [36, 39]. GL as well as its derivatives was also capable of neutralizing the secretion of these proinflammatory chemokines [36, 39].

On the other hand, GA was capable of decreasing the secretion of vascular endothelial growth factor (VEGF), intercellular cell adhesion molecule 1 (ICAM-1), granulocyte-macrophage colony-stimulating factor (GM-CSF), and human growth-regulated oncogene/keratinocyte chemoattractant (GRO/KC) in alcoholic hepatitis rats' models (Figure 1 and Table 1) [40]. GA was also capable of inhibiting phospholipase A2/arachidonic acid (PLA2/ARA) (Table 1) pathway metabolites, like prostaglandin-E 2 (PGE2) or prostacyclin 2, thromboxane 2 (TXA_2_), and leukotriene B4 (LTB4) (Figure 1) [41, 42]. It was stipulated that the anti-inflammatory response to GL and GA was a result of direct binding the molecules to cell membrane constituents like lipocortin I (LC-1) or to enzymes such as PLA2 (Table 1), which is the prime enzyme in the arachidonic acid metabolic pathway (Figure 1) [36]. GA ominously decreased the concentration of ICAM-1 as well as matrix metalloproteinase-9 (MMP-9) (Figure 1) [41, 43]. Furthermore, it augmented the actions of Superoxide dismutase (SOD) and glutathione peroxidase (GSH-Px), as well as the secretion of p-Akt and p-ERK (Figure 1) [41, 44].

GL and GA efficiently blocked the generation of free radicals in LPS-treated Raw264.7 macrophage models [41]. They also decreased the configuration of the LPS-TLR-4/MD-2 complexes, leading to the blockade of homodimerization of TLR-4 (Figure 1) [45, 46]. Thus, GA was able to regulate the TLR-4/MD-2 complex at the receptor level, resulting in the inhibition of LPS-induced triggering of signaling cascades as well as cytokine generation [45]. This signifies that GA blocked inflammatory responses as well as regulated innate immune responses [45, 47].

Furthermore, GA inhibited the stimulation of signal transducers and activators of transcription-3 (STAT-3), decreased the upregulation of ICAM-1 as well as P-selectin secretion, decreased the configuration of poly-adenosine diphosphate-ribose (pADR) and nitrotyrosine (NTS), and decreased polymorphonuclear neutrophil infiltration (PMN) (Figure 1 and Table 1) [45–47]. Moreover, GA elicited broad anti-inflammatory actions via its interaction with the lipid bilayer resulting in the decrease of receptor-mediated signaling [45, 46]. GA was capable of blocking the lytic pathway of the complement system as well as averted tissue injury triggered by membrane attack complexes [45].

## 5. Dendritic Cells

Dendritic cells (DCs) are a group of bone-marrow-derived cells found in blood, tissues, and lymphoid organs [48–50]. These cells initiate and control immune responses that are affected by numerous factors like origin, phenotype, and maturation status [48–50]. Their prime function is to bridge the innate as well as adaptive immune systems [48–50]. DCs were able to accelerate allogeneic T-cell proliferation *in vitro* [4]. A study revealed that only a minute quantity of DCs was enough to trigger an allogeneic mixed lymphocyte reaction (MLR) [4, 48]. Studies have demonstrated that DCs are the most crucial antigen-presenting cells (APCs) associated with the uptake, processing, transport, and presentation of antigens to CD4^+^ and CD8^+^ T-cells [4, 49, 51].

**Figure 2:**
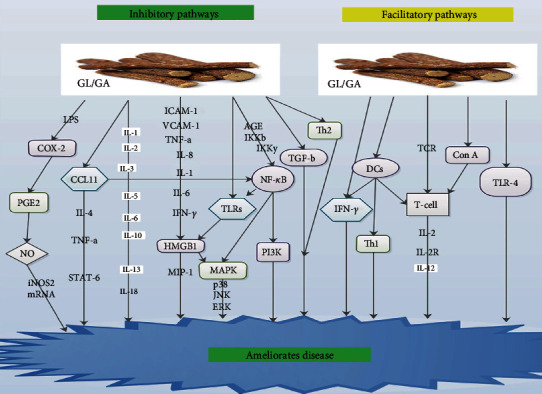
Shows the inhibitory and facilitatory pathways via which GL and GA ameliorate disease.

Also, DC subsets are capable of triggering or inhibiting immune responses via the secretion of different costimulatory molecules and cytokines [4, 52]. DCs were able to trigger as well as target naive T-cells to differentiate into T-helper (Th)1 or T-helper (Th)2 cells [4, 53]. Thus, DCs have potential immunomodulatory therapeutic targets for some pharmacological compounds [4, 10]. Bordbar et al. demonstrated that DCs treated with GL were capable of influencing T-cell differentiation toward Th1 subset (Figure 2) (Table 1) [4]. Abe et al. also observed the upregulation of IL-10 expression by liver DCs [54]. Hua et al. established that GL was capable of augmenting IL-10 production in DC2.4 cell line (Figure 2) [55]. A current study demonstrated that GL was capable of augmenting IL-10 production along with IFN-*γ* in MLR [4]. On the other hand, Bhattacharjee et al. exhibited that GA was capable of blocking the expression of the Th2, IL-10, and TGF-*β* from the splenocytes of infected mice (Figure 2) [25].

## 6. Nuclear Factor-*κ*B

The nuclear factor-*κ*B (NF-*κ*B) family is made up of five groups such as NF-*κ*B1, which comprise of p50/p105 with p50 as the precursor, NF-*κ*B2 which comprise of p52/p100 with p52 as the precursor, Rel A with p65 as the precursor, Rel B with p68 as the precursor, and c-Rel with p75 as the precursor [56, 57]. Almost all the groupings are capable of preserving homodimeric as well as heterodimeric complexes [56]. Nevertheless, the most predominant-stimulated form of NF-*κ*B is the heterodimer p50-p65, which has the transactivity territory obligatory for gene modification [58–60]. In most cells, NF-*κ*B exists as a latent, inactive, I*κ*B bound complex in the cytoplasm [56]. Nevertheless, upon stimulation by extracellular stimuli, NF-*κ*B promptly translocates to the nucleus and triggers gene release [56, 61].

I*κ*B kinase (IKK) is a large multisubunit protein kinase active via numerous signal pathways [56]. The IKK complex when triggered results in the phosphorylation or degradation of I*κ*B*α* leading to the expression of NF-*κ*B [56]. NF-*κ*B then translocates to the nucleus and triggers the transcription of numerous *κ*B-dependent genes, such as iNOS as well as Th1 cytokines [56]. Thus, some pathogens are capable of blocking the action of NF-*κ*B via the inhibition of the degradation of I*κ*B during infection [56]. Also, in macrophages, the MAPK cascade and the NF-*κ*B pathway are the key pathways via which modulation of inflammation as well as host defense occurs [56].

Ukil et al. demonstrated that the kinase properties of IKK were triggered in cells that were stimulated with GA via a mechanism that most probably involves upregulatory signaling pathways [56]. They however did not observe any influence of GA on IKK activity when GA was added directly to the assay mixture containing IKK immunoprecipitated from normal macrophages (Table 1) [56]. An earlier study revealed that GA influenced the inhibitory interaction between NF-*κ*B, which is a fundamental modifier to IKK*β* and IKK*γ* (Figure 2) [62]. Another study indicated that GA inhibited one of the essential upregulatory kinases like NF-*κ*B-inducing kinase, PI3K, or MAPK in the signaling pathway (Figure 2) [63].

GL was capable of treating coxsackievirus B3- (CVB3-) triggered myocarditis via the blockade of CVB3-triggered NF-*κ*B activity via the inhibition of NF-*κ*B inhibitor I*κ*B (Table 1) [20, 64]. Wang and Du revealed that pretreatment with GL substantially inhibited the facilitation of NF-*κ*B p65 protein secretion, in methotrexate-stimulated enteritis (Table 1) [6]. Cherng et al. showed that GL blocked NF-*κ*B secretion, averted DNA damage, and accelerated DNA repair (Table 1) [65]. Feng et al. demonstrated that GA safeguards advanced glycation end-product- (AGE-) stimulated endothelial dysfunction via blockade of the receptor for AGE/NF-*κ*B signaling pathway (Figure 2 and Table 1) [66].

## 7. Chemokines

Chemokines are a family of molecules associated with the trafficking of leukocytes in normal immune surveillance and recruitment of inflammatory cells in host defense [67–69]. They are made up of over 40 groups, which are classified into four classes founded on the sites of essential cysteine residues like C, CC, CXC, and CX3C [67]. GL was capable of subduing the H5N1-triggered generation of CXCL10, and CCL5 resulting in the blockade of H5N1-triggered apoptosis [20, 70]. Michaelis et al. demonstrated that 100 mg/ml of GA drastically blocked secretion of CXCL10, and CCL5 at the mRNA and the protein levels (Table 1) [30]. Augmented CXCL10 levels were observed in patients with H5N1, and the elevated levels of CXCL10 were associated with poor prognosis (Table 1) [30, 71].

CCL11 known as eotaxin 1 was primarily detected as the prime eosinophil chemoattractant in the lung lavage fluid after allergic exposure in guinea pigs [39]. Subsequently, it was cloned for further studies [39, 72, 73]. Several studies have demonstrated that numerous types of cells, such as lung or dermal fibroblasts, as well as lung or bronchial epithelial cells are capable of producing eotaxin 1 [39, 74, 75]. Studies further revealed that the production of eotaxin 1 was triggered by IL-4 and inhibited by IFN-*γ* [39, 74, 75]. It was also observed that eotaxin 1 facilitated the infiltration of eosinophils into allergic inflammatory sites [39, 74, 75].

Matsui et al. indicated that GL may be capable of modulating chemokine generation via the posttranscriptional level such as protein expression or mortification [39]. They demonstrated that GL derivatives had inhibitory effects on eotaxin 1 generation via TNF-*α* as well as IL-4 induction in lung fibroblasts (Figure 2) [39]. Studies have shown that induction of IL-4 and TNF-*α* in combination synergistically accelerated the generation of eotaxin 1 via the triggering of transcriptional factors like STAT-6 and NF-*κ*B (Figure 2) [76–78]. GL and its derivatives thus blocked eotaxin 1 production at protein or mRNA secretary levels (Table 1) [39, 76].

## 8. Interferons

Interferons (IFNs) are a family of broad-spectrum antiviral glycoproteins expressed by cells upon attack by viruses. They are often involved in numerous immune responses as triggers, modulators, and effectors of both innate as well as adaptive immune systems during viral infections [79, 80]. They have the ability of blocking viral replication and are often the most prominent cytokines produced during viral infections [79, 80]. IFN-*γ*, which is expressed by lymphocytes, has been implicated in the secretion of histocompatibility antigen as well as immune modifications [6, 81]. Studies have demonstrated that IFN-*γ* was capable of facilitating the endotoxin-stimulated generation of NO in murine macrophages [79, 80].

Studies have shown that IFN with or without adenine arabinoside was capable of curing hepatitis B patients [1, 82, 83]. IFNs were capable of reducing the level of either DNA polymerase or hepatitis B surface antigen in hepatitis patients [1, 84]. Furthermore, GL was capable of facilitating IFN-*γ* production in human T-lymphocytes [85, 86]. Also, GL was capable of inducing the production of IFN in mice, which was preceded by stimulation of macrophages as well as the increase of NK activity [87, 88]. Bhattacharjee et al. demonstrated that splenic expression of IFN-*γ*, TNF-*α*, and IL-12 elevated after GA treatment. Wu et al. also demonstrated that GL drastically decreased inflammatory via IFN-*γ* (Figure 2) [89]. They concluded that blockade of the IFN-*γ* signaling pathway may be linked to anti-inflammatory effects of GL in enteritis [89].

## 9. Cyclooxygenase and Prostaglandins

COX-1 and COX-2 are the main cyclooxygenase (COX) isoenzymes, which catalyze the formation of prostaglandins, thromboxane, and levuloglandins [90]. Prostaglandins are autocoid facilitators that influence practically all recognized physiological as well as pathological activities via their reversible communication with G-protein attached membrane receptors [90]. Amongst the COX isoenzymes, COX-2 was more inducible with low secretory levels in most tissues under normal circumstances [6, 91]. It was established that numerous cell types such as vascular smooth muscle cells, endothelial cells, mononuclear macrophages, and fibroblasts were capable of secreting COX-2 up to about 8-10-fold the normal level when stimulated by proinflammatory cytokines [6, 92].

It was further observed that augmentation of COX-2 levels resulted in the generation as well as buildup of prostaglandin inflammatory factors, facilitating inflammatory responses as well as tissue damage [6, 91]. Studies have shown that oversecretion of COX-2 facilitated cell proliferation, blocked apoptosis, and blocked immune responses, resulting in abnormal modulation of the balance between proliferation and apoptosis [6, 91, 92]. Bhattacharjee et al. demonstrated that a robust antileishmanial protection was observed via the modulation of macrophage-secreted COX-2-determined PGE2 levels [25]. Also, Leishmania organisms were capable of using immune modulators like TGF-*β*, IL-4, and arachidonic acid metabolites to inhibit macrophage functions and facilitated the organism's survival within the host [93].

PGE2 biosynthesis comprises two successive enzymatic reactions [25]. The first one is a rate-limiting step involving the COX enzyme, while the second is a precise PGE synthesis step [25]. In pathophysiological processes, the inducible isoform of COX-2 was capable of modulating PGE2 production while COX-1 was principally copied [25, 94, 95]. Studies have shown that augmented level PGE2 was capable of modulating several immune responses via mechanisms involving the blockade of Th1 cytokines like IL-2, IL-12, and IFN-*γ*, as well as inhibition of phagocytosis and lymphocyte proliferation [25, 96, 97]. Thus, PGE2 ability to modulated immune response is champion by Th1- or Th2-associated lymphokines [25, 96].

It was established that GA was capable of blocking PGE2 synthesis via blockade of COX-2 resulting in concurrent augmentation NO production through enhancement of iNOS2 mRNA secretion in Leishmania-infected macrophages (Figure 2 and (Table 1) [25]. Wang and Du demonstrated that pretreatment with GL significantly blocked the facilitation of COX-2 activity in methotrexate-triggered enteritis [6]. Cherng et al. also demonstrated that GL was able to block COX-2 secretion, inhibited DNA damage, and promoted DNA repair (Table 1) [65]. Ni et al. observed an upsurge in COX-2 secretion in lung tissues after introducing LPS in their experiment, which was subsequently decreased in a dose-dependent manner after GL pretreatment (Figure 2 and Table 1) [98].

## 10. Interleukins

Interleukin belongs to a group of cytokines, which are perhaps the most essential messenger molecules generated by leukocytes to modulate the biological activities of target cells via autocrine or paracrine means [99]. Several groupings of ILs have been identified [99]. Notable amongst them are IL-1, IL-2, IL-3, IL-5, IL-6, IL-10, IL-12, IL-13, and so many others [3, 8, 99–101]. Although most of the ILs are influenced by GL and GA, IL-12 is the most influential. IL-12 is a heterodimeric cytokine produced primarily by macrophages and monocytes [8]. Its key function is the modulation of cytokines as well as T-cell subsets [8]. A study revealed that a deficiency in endogenous IL-12 production influenced the progression of immunodeficiency in HIV-infected patients [8, 102]. Studies have proven that IL-12 salvaged numerous activities of cells infected with HIV [8, 103].

Several studies have demonstrated that IL-12 was capable of influencing T-cells and natural NK cells resulting in cell proliferation, cytolytic activities, and triggering of IFN-*γ* [8, 104]. Studies further revealed that the polarization of the T helper response to a Th1-dominant form via IL-12 was accelerated by IFN-*γ* resulting in the blockade of IL-4 production [8, 100, 101]. GA was capable of blocking IL-1*β*, IL-3, IL-5, IL-6, IL-10, IL-12 subtypes, IL-13 (Figure 2), eotaxin, and TNF-*α* expression (Table 1) [8, 104–106]. GL was also capable of accelerating the proliferation of lymphocytes and acted as a facilitator of the late signal transduction of T lymphocytes for IL-2 generation (Table 1) [4, 106].

Zhang et al. also indicated that GL facilitated TCR-mediated T-cell proliferation by selectively influencing the late signal transduction for IL-2 generation as well as IL-2R secretion [9]. They further indicated that GL exhibited two separate activities on immature thymocytes resulting in the facilitation of IL-2 generation on one hand and blocked growth response on the other [9, 107]. A hepatitis study revealed that IL-4 was capable of stimulating STAT6, which in turn stimulated eotaxin secretion as well as triggered IL-5 secretion [40, 108]. Wang and Du established that GA was capable of relieving methotrexate-stimulated upsurge of TNF-*α*, IL-1*β*, and IL-6 levels, as well as elevated IL-10 levels, in rats with enteritis (Table 1) [6]. GL was able to facilitate the IL-10 production by hepatic dendritic cells in mice with hepatitis (Table 1) [76].

Studies have proven that IL-10 is a well-known anti-inflammatory cytokine [40, 109, 110]. It was capable of modulating STAT3 in hepatocytes as well as macrophages/Kupffer cells [40, 109, 110]. A study revealed that GA was capable of accelerating LPS-triggered IL-12 generation by peritoneal macrophages (Table 1) [4, 8]. Its optimal effect on IL-12 gene secretion was linked to an upsurge in NF-*κ*B modulation [4, 8]. Dai et al. demonstrated that GL accelerated both IL-12 mRNA buildup as well as protein expression by peritoneal macrophages in response to LPS [8]. They indicated that the priming influence of GL on IL-12 generation did not depend on IFN-*γ* or GM-CSF [8]. Thus, they also affirmed that the facilitation of IL-12 p40 mRNA secretion by GL may be via the modulation of NF-*κ*B [8].

Yoshida et al. demonstrated that GL was able to block the upsurge in serum levels of IL-18 in LPS/D-galactosamine-induced liver injury (Figure 2 and Table 1) [111]. Thus, GL blocked the generation of IL-18 in this model [111]. They also observed fewer IL-18-positive infiltrating cells after the introduction of GL [111]. Also, GL was capable of blocking the infiltration of neutrophils and macrophages in liver injury [111]. Furthermore, GL-stimulated decrease in immunoreactive IL-18 was probably due to blockade of cell infiltration in the liver [111]. GL was able to inhibit an upsurge in alanine aminotransferase activity when exogenous IL-18 was administered in mice treated with LPS/D-galactosamine [111]. Thus, GL blocked IL-18-mediated inflammatory response in the pathogenesis of liver injury [111]. Nakanishi et al. demonstrated that IL-18 was capable of triggering gene secretion as well as the synthesis of TNF-*α*, IL-1, FAS ligand, and many chemokines [112].

## 11. Mitogen-Activated Protein Kinase

Mitogen-activated protein kinase (MAPK) signal transduction pathways are linked with cell proliferation, differentiation, apoptosis, and angiogenesis [6]. Specifically, the p38 mitogen-activated protein kinase (p38MAPK) signal transduction pathway modulates stress responses, like inflammation as well as apoptosis [6, 113]. Studies have shown that LPS as well as other factors is capable of triggering the MAPK pathways resulting in the secretion of many inflammatory mediators via complex signal conduction pathways, which facilitates inflammation [6]. Furthermore, the modulation of p38MAPK was observed in various transduction pathways, which in turn stimulated many transcription factors as well as mediated a variety of biological activities [6, 114].

Wang and Du demonstrated that pretreatment with GA remarkably inhibited the facilitation of p38MAPK in methotrexate-stimulated enteritis (Table 1) [6]. They concluded that the anti-inflammatory actions of GA were probably linked to p38MAPK signaling (Figure 2) [6]. Also, studies have shown that GA lessens glycative stress in the kidneys of diabetic mice via the blockade of p-p38MAPK [115, 116]. It was further established that GA was capable of blocking the modulation of JNK, p38 protein, and ERK (Figure 2 and Table 1) in bone marrow-derived macrophages (BMMs) [45].

## 12. Nitric Oxide

Nitric oxide (NO) is a radical messenger molecule generated by the enzyme nitric oxide synthase (NOS) [117–119]. So far, only three isoforms of NOS have been identified. Amongst the three, only two of them, NOS in neurons (nNOS) and in the endothelial cells of blood vessels (eNOS), are intensely secreted [117–120]. These two are capable of producing only minute quantities of NO, which is sufficient to trigger cellular signaling in stress conditions.

Studies have shown that NO in an inflammatory mediator is capable of modulating innate immunity as well as pathophysiology of many infectious diseases [117, 121, 122]. The third kind of NOS is the inducible nitric oxide synthase (iNOS) [117, 119].

Studies have further proven that iNOS generates NO in hepatocytes as well as macrophages [117, 119, 121, 122]. The stimulation of iNOS is modulated via a posttranscriptional mechanism that is mediated by antisense transcripts (asRNAs) [117, 122]. Several studies have shown that the asRNAs are transcribed from the iNOS gene and interact with iNOS mRNA to stabilize the same iNOS mRNA [122, 123]. Studies have demonstrated that the iNOS is triggered by cytokines like IFN-*γ* and TNF-*α*, which in turn produce large quantities of NO [117, 119, 121, 123]. It is well proven that NO generated by iNOS was capable of triggering an inflammatory liver damage [117, 124].

Studies have demonstrated that concanavalin A (Con A) was capable of triggering the stimulating the T-cells in mice and induced the secretion of proinflammatory cytokines associated with the progression of hepatitis (Figure 2) [119, 125]. Furthermore, GL was capable of inhibiting Con A-stimulated mouse liver damage without influencing the generation of IFN-*γ* and TNF-*α* [119, 126]. Tsuruoka et al. demonstrated that GL blockade of liver damage was via the inhibition of iNOS mRNA as well as its protein secretion (Table 1) [119]. Thus, GL inhibited iNOS mRNA and protein in Con A-stimulated hepatitis [119]. Also, GL was capable of blocking the secretion of iNOS mRNA stimulated by carbon tetrachloride in hepatic tissue (Table 1) [119, 127].

## 13. Toll-Like Receptors

Toll-like receptors (TLRs) are sensors for pathogen-associated molecular patterns (PAMPs) [128]. TLRs are capable of modulating several immune responses, especially during the infectious process [128]. Several studies have shown that the secretion of TLR-3, TLR-4, TLR-7, TLR-9, and TLR-10 genes from hepatic tissue was upregulated in some viral infection models [129, 130], and GA or GL is capable of inhibiting these receptors (Table 1) [131–134]. It was established that the TLR-4 pathway comprises of two dissimilar signaling pathways such as the myeloid differentiating primary response gene 88- (MyD88-) dependent as well as the MyD88-independent pathway [135, 136]. It was further revealed that stimulation of the MyD88-dependent pathway led to the generation of proinflammatory cytokines via triggering of NF-*κ*B, while the stimulation MyD88-independent pathway led to the generation of type 1 IFNs [135, 136].

A study revealed that TLR-4 was the fundamental receptor of the innate immune signaling responses to influenza virus as well as other respiratory viruses [137]. Several studies have shown that the TLR-4 was more associated with respiratory syncytial virus and human papillomavirus infections [129, 138, 139]. Shi et al. revealed that TLR-4 gene deficiency was not associated with the downregulation of virus titer in the liver during MHV-A59 infection [129]. They observed that in MHV-A59 infection, the HMGB1-TLR-4 axis utilizes proinflammatory activities without directly influencing virus replication [129].

A study demonstrated that GA was not capable of influencing TLR-4 gene secretion during viral infection [129]. Nevertheless, the secretion of the TLR-4 gene facilitated MHV-stimulated hepatic inflammation injury as well as determined HMGB1 secretory levels in the serum (Figure 2) [129]. Several studies have proven that pretreatment with a TLR-4 inhibition agent reduced the HMGB1 levels from virus-infected cells via the TLR4-NF-*κ*B pathway (Figure 2) [129, 139, 140]. Studies further revealed that the inactivation of NF-*κ*B led to a reduced expression of different proinflammatory cytokines like IL-1*β*, IL-6, TNF-*α*, and HMGB1 (Figure 2) [129, 139, 140]. GL was capable of blocking porcine epidemic diarrhea virus infection, as well as reduced proinflammatory cytokine expression via the HMGB1/TLR4-p38MAPK pathway (Figure 2 and Table 1) [141].

## 14. High-Mobility Group Box 1

High-mobility group box 1 (HMGB1) protein is a nuclear protein that functions as an architectural chromatin-binding factor [142, 143]. HMGB1 is the prime signal during tissue damage usually involving necrotic and apoptotic cells [142]. Furthermore, HMGB1 performs dual functions in the nucleus and the cytoplasm [142]. Also, extracellular HMGB1 facilitates both local as well as systemic responses in the organism [142]. These responses often include inflammation, modulation of innate as well as adaptive immunity [142, 143]. Several studies have demonstrated that HMGB1 is secreted by monocytes, macrophages, neutrophils, platelets, and dendritic and NK cells [142, 144].

Several studies have shown that HMGB1 induces macrophages, monocytes, and neutrophils to secrete proinflammatory cytokines like TNF-*α*, IL-1, IL-6, IL-8, and MIP-1 via p38- and JNK MAPK-dependent pathways (Figure 2) [145, 146]. It was established that HMGB1 was passively secreted by damage alveolar endothelial cells or macrophages during virus-mediated cytolysis [145]. Once expressed, extracellular HMGB1 was capable of mediating injurious pulmonary inflammatory response like neutrophil infiltration, derangement of epithelial barrier, lung edema, and lung injury [145, 147]. These injurious pulmonary inflammatory responses subsequently result in respiratory failure as well as death [147].

Also, human microvascular endothelial cells are capable of secreting ICAM-1, vascular adhesion molecule–1 (VCAM-1), proinflammatory cytokines like TNF*α*, IL-8, and chemokines in response to HMGB1 activation (Figure 2) [145, 148]. This means that HMGB1 was capable of disseminating inflammatory response in the endothelium during infection or injury [145]. Chemotactic as well as mitogenic actions of HMGB1 depends on its association with the receptor of advanced glycation end products (RAGE) [142, 149]. GL was capable of blocking the chemoattractant as well as mitogenic activities of HMGB1 (Table 1) [142].

GL was capable of binding to both HMG boxes of HMGB1 in both NMR and fluorescence studies without altering their secondary structure, which was observed as an absence of changes in CD spectra [142]. It was further established that amino acids interacting with GL clusters at the junction of both arms of the classical L-shape fold of both HMG boxes in chemical-shift perturbation experiments [142]. Furthermore, the binding sites for GL on the HMG boxes partly overlap with the DNA binding sites, shielding residues like R23, which is recognized to be crucial for DNA binding [142, 150]. Nevertheless, the RAGE-binding surface on HMGB1 was characterized with the stretch of basic amino acids between box B and the acidic tail and did not match with the binding surfaces of GL [142, 149].

Influenza type A, B, and C viruses are responsible for influenza infection (“flu”) [145]. This infection is often depicted with massive virus replication as well as excessive inflammation [145]. Studies have shown that influenza viruses are capable of infecting monocytes and macrophages resulting in the stimulation of proinflammatory cytokines like TNF-*α*, IL-1, IL-6, IL-8, IFN-*α*, and chemokines in infected areas (Figure 2) [145, 151]. Moisy et al. demonstrated that HMGB1 binds to the nucleoprotein section of influenza ribonucleoproteins (vRNPs) freely in the company of viral RNA *in vitro* and interacts with the viral nucleoprotein VCAM-1 in infected cells [152]. They revealed that HMGB1 was capable of facilitating viral growth as well as augmented the transcription or replication activity of the viral polymerase in HMGB1-depleted cells [152]. Thus, HMGB1 binding to DNA was a prerequisite for the augmentation of influenza virus replication [152]. Therefore, GA and GL may be capable of treating influenza viral infection via the HMGB1-TNF-*α* pathway (Figure 2). Further studies should focus on this pathway.

HMGB1 was able to trigger necrotic cell death resulting in abundant budding of West Nile (WN) progeny virus particles at higher infectious doses [145, 153]. Furthermore, HMGB1 mediated in injurious inflammatory response resulting in the pathogenesis of WN encephalitis [145, 153, 154]. Besides WN viruses, other viruses like the salmon anemia virus were capable of triggering necrotic cell death of infected cells, leading to simultaneous HMGB1 expression [145, 154]. GL and GA may be potential treatment options for WN viral via HMGB1. Further studies are warranted in this direction.

Studies have shown that an increase in proinflammatory cytokines like IL-1, IL-6, TNF-*α*, and IFN-*γ* may trigger the expression of HMGB1 from innate immune cells in SARS patients (Figure 2) [145, 155]. Thus, further studies on GL/GA-HMGB1 axis are needed to elucidate their potential role in the treatment for patients with coronavirus disease-19 in the current SARS-coronavirus pandemic. Acute viral hepatitis is caused by hepatitis A, B, C, and D viruses. Their pathogenesis is often depicted with acute necrosis of hepatocytes, inflammation, and followed by fibrosis as well as cirrhosis [145, 156]. HMGB1, passively secreted by necrotic hepatocytes, may stimulate tissue macrophages especially Kupffer cells to express proinflammatory cytokines during an acute infection [145]. Thus, HMGB1 alone or in combination with other proinflammatory cytokines may cause chronic liver damage in hepatitis patients [145]. GL and GA are potential treatment options for chronic viral hepatitis. Further studies are warranted on HMGB1 and/or GL/GA axis.

## 15. Conclusion

GL and GA are able to block the secretion of IL-1*β*, IL-3, IL-4, IL-5, IL-6, IL-10, IL-12, IL-13, eotaxin, and TNF-*α* expression. This means that GL and GA are capable of inhibiting cytokine storms elicited during various infectious diseases most especially viral diseases. GL and GA drastically decreased inflammation via IFN-*γ*, which means that GL and GA have very crucial antiviral properties. Also, GA decreased the secretion of VEGF, MCP-1, GM-CSF, and GRO/KC in alcoholic hepatitis rats' models. GA was capable of blocking the modulation of JNK, p38 protein, and ERK in BMMs. Further studies on GL/GA-HMGB1 axis are needed to elucidate their potential role in the treatment for patients with coronavirus disease-19 in the current SARS-coronavirus pandemic.

## Figures and Tables

**Table 1:** Shows the explicit effect of GL or GA on various immune/inflammatory factors.

Immune/inflammatory factors	Type	Effect of GL/GA	Citations
Inflammation	VEGF	Inhibitory	[40]
	ICAM-1	Inhibitory	[40]
	GM-CSF	Inhibitory	[40]
	GRO/KC	Inhibitory	[40]
	PLA2/ARA	Inhibitory	[36, 41, 42]
	MMP-9	Inhibitory	[41, 43]
	STAT-3	Inhibitory	[45–47]
	STAT-6	Inhibitory	[45–47]
	pADR	Inhibitory	[45–47]
	NTS	Inhibitory	[45–47]
	PMN	Inhibitory	[45–47]
	SOD	Facilitatory	[41, 44]
	GSH-Px	Facilitatory	[41, 44]
	TGF-*β*	Facilitatory	[25]
Dendritic cells (DCs)	T-cell	Facilitatory	[4]
	Th1	Facilitatory	[4]
	Th2	Facilitatory	[25]
Nuclear factor-*κ*B	—	Inhibitory	[6, 20, 62–65]
	IKK	Inhibitory	[56]
Chemokines	CXCL10	Inhibitory	[20, 30, 70, 71]
	CCL5	Inhibitory	[20, 30, 70, 71]
	CCL11	Inhibitory	[39, 76–78]
Interferons	IFN-*γ*	Facilitatory	[85–89]
Cyclooxygenase	COX-1	—	—
	COX-2	Inhibitory	[25, 65, 98]
Interleukins	IL-1	Inhibitory	[8, 104–106]
	IL-2	Facilitatory	[4, 9, 106]
	IL-3	Inhibitory	[8, 104–106]
	IL-4	Inhibitory	[8, 104–106]
	IL-5	Inhibitory	[8, 104–106]
	IL-6	Inhibitory	[8, 104–106]
	IL-10	Inhibitory	[8, 104–106]
	IL-12	Inhibitory	[4, 8, 104–106]
	IL-13	Inhibitory	[8, 104–106]
	IL-18	Inhibitory	[8, 104–106, 111]
Mitogen-activated protein kinase	—	Inhibitory	[6, 115, 116, 141]
	p38MAPK	Inhibitory	[6, 115, 116]
	ERK	Inhibitory	[45]
	JNK	Inhibitory	
Nitric oxide	iNOS	Inhibitory	[45]
	eNOS	—	—
	nNOS	—	—
Toll-like receptors	TLR-3	Inhibitory	[131–134]
	TLR-4	Inhibitory	[131–134, 141]
	TLR-7	Inhibitory	[131–134]
	TLR-9	Inhibitory	[131–134]
	TLR-10	Inhibitory	[131–134]
High-mobility group box 1	—	Inhibitory	[141, 142]

## Data Availability

No data was used in this paper.

## Abbreviations

asRNAs: Antisense transcripts
APCs: Antigen-presenting cells
BMMs: Bone marrow-derived macrophages
COX: Cyclooxygenase
GC: Carbenoxolone
CVB3: Coxsackievirus B3
Con A: Concanavalin A
DCs: Dendritic cells
GM-CSF: Granulocyte-macrophage colony-stimulating factor
GL: Glycyrrhizin
GA: Glycyrrhetinic acid
HMGB1: High-mobility group box 1
GRO/KC: Human growth-regulated oncogene/keratinocyte chemoattractant
HIV: Human immunodeficiency virus
ICAM-1: Intercellular cell adhesion molecule 1
IKK: I*κ*B kinase
IFNs: Interferons
IL: Interleukin
iNOS: Inducible nitric oxide synthase
MAPK: Mitogen-activated protein kinase
p38MAPK: p38 mitogen-activated protein kinase
MLR: Mixed lymphocyte reaction
mRNA: Messenger RNA
NK: Natural killer
NO: Nitric oxide
NF-*κ*B: Nuclear factor-*κ*B
NTS: Nitrotyrosine
PLA2: Phospholipase A2
pADR: Poly-adenosine diphosphate-ribose
PMN: Polymorphonuclear neutrophil infiltration
TNF-*α*: Tumor necrotizing factor-*α*
PGE2: Prostaglandin-E 2
vRNPs: Ribonucleoproteins
TLRs: Toll-like receptors
Th: T-helper
SARS: Severe acute respiratory syndrome
STAT: Signal transducer and activator of transcription
VCAM-1: Vascular adhesion molecule–1
VEGF: Vascular endothelial growth factor
WN: West Nile.
